# Untargeted LC-QToF-MS profiling of the soybean (*Glycine max*) root-soil continuum reveals spatial compartmentalisation of putative exudate, defence, and iron-acquisition metabolite signatures

**DOI:** 10.3389/fpls.2026.1940810

**Published:** 2026-09-21

**Authors:** Kirti Suman, Hirudhaya Ravi, Pushpendra Sharma, Mahaveer P. Sharma, Minakshi Grover, Rajeev Kaushik

**Affiliations:** 1 Division of Microbiology, ICAR-Indian Agricultural Research Institute, New Delhi, India; 2 ICAR-National Soybean Research Institute, Indore, India

**Keywords:** isoflavonoids, LC-QToF-MS, rhizosphere, root exudates, siderophores, soybean, untargeted metabolomics

## Abstract

**Introduction:**

Compartment-resolved metabolite baselines for legumes grown under field conditions are notably scarce. Most root exudate data are obtained from hydroponic or pot systems using targeted assays. These methods fail to accurately represent the soil-borne and microbially derived chemical environments surrounding roots *in situ*. Furthermore, no such baseline has been established for soybeans grown on the calcareous Vertisols of central and western India, where phosphorus, Zn, and Fe availability are the primary constraints.

**Methods:**

We employed untargeted liquid chromatography-quadrupole time-of-flight mass spectrometry (LC-QToF-MS) to analyze four compartments of a field-grown soybean system in Hadoti, southeastern Rajasthan, India. These included non-rhizospheric bulk soil (NRBS), rhizospheric soil (RS), surface-sterilized (SRS), and unsterilized (SRU) roots, with three replicates each. We aimed to identify compartment-specific metabolic signatures and potential plant-microbe interaction mediators. As data were obtained at the MS1 level without tandem-MS confirmation, all annotations are putative, in accordance with Metabolomics Standards Initiative Levels 2-3.

**Results:**

Principal component analysis distinguished the root compartments, whereas the soil compartments were differentiated along the first component with overlapping confidence regions. Set analyses revealed 43 features exclusive to RS and 120 features exclusive to SRU. Soil chemistry differed markedly between NRBS and RS: available phosphorus, potassium, zinc, and manganese were lower in the rhizosphere, whereas exchangeable aluminium was approximately 15 times higher and was the sole element accumulating in the root zone. Among the ecological features, root colonization correlated with reduced abundance of jasmonic acid features and increased abundance of isoflavonoid features (including daidzein) and microbial siderophores. Although substantial, these differences did not withstand false discovery rate correction and are reported as candidate observations.

**Discussion:**

The observed patterns align with established models of nutrient mobilization, symbiotic signalling, and iron competition. However, targeted MS/MS validation and independent quantification are required before drawing mechanistic conclusions. The dataset serves as a hypothesis-generating baseline and provides a prioritized set of candidate features for subsequent targeted investigations of soybean root-microbe chemical interactions.

## 1 Introduction

The rhizosphere, defined as the soil zone directly influenced by the chemical and biological activities of living roots, is one of the most metabolically active interfaces in terrestrial ecosystems (Philippot et al., 2013). This zone facilitates interactions between host-plant biochemistry and the metabolic processes of the soil microbiome (Vandenkoornhuyse et al., 2015). Unlike bulk soil, which is primarily shaped by parent material, weathering, and long-term organic matter turnover, rhizosphere soil is continuously modified by the release of photosynthetically fixed carbon, organic acids, secondary metabolites and volatile compounds (Bais et al., 2006). Root exudation is now recognised as a resource-intensive process rather than a passive by-product of root activity, and it plays a crucial role in nutrient acquisition, abiotic stress mitigation, and assembly of the root-associated microbiome (Sasse et al., 2018).

In legumes such as soybean (*Glycine max* (L.) Merr.) chemical interactions among roots, soil, and microbes are crucial for nitrogen management and sustainable production, supporting biological nitrogen fixation (Peoples and Craswell, 1992; Peoples et al., 1995). In central India, soybean is primarily cultivated as a rainfed monsoon crop on Vertisols, commonly known as “black cotton soils.” These soils are characterised by smectitic (montmorillonitic) clays and exhibit pronounced shrink-swell behaviour owing to seasonal moisture fluctuations (Deckers et al., 2001). Although these soils have a high cation-exchange capacity, they pose agronomic challenges, including alkalinity, poor internal drainage, and nutrient depletion under continuous cropping (Shinogi et al., 2026). A persistent issue with these calcareous soils is the limited bioavailability of phosphorus, zinc, and iron (Janati et al., 2023). Under alkaline conditions, inorganic phosphorus tends to precipitate as calcium phosphates, whereas Fe(III) and Zn become sparingly soluble, thereby restricting root development and nodulation.

The rhizosphere priming effect (RPE) partially mediates root responses to environmental constraints by altering the decomposition rate of native soil organic matter through the activity of living roots and rhizodeposition (Kuzyakov, 2010; Huo et al., 2017). Labile carbon derived from roots can enhance microbial activity, which in turn accelerates the mineralization of soil organic matter and the mobilisation of nutrients via extracellular enzymes (Zhu et al., 2018). Consequently, elucidating the specific metabolites released into and produced within the rhizosphere has both ecological and practical significance, particularly in developing exudate-derived biostimulants to enhance nutrient acquisition and symbiosis (Calvo et al., 2014; Aloo et al., 2022).

Analytical methods that simultaneously capture numerous metabolites are required to address this chemical complexity. Targeted assays effectively quantify a predefined set of compounds; however, they inherently overlook most metabolites. In contrast, untargeted high-resolution LC-MS can detect thousands of features in a single analysis, making it well suited for generating hypotheses about compartment-specific chemistry (van Dam and Bouwmeester, 2016; Pang et al., 2021). A recognised limitation of untargeted MS1 workflows is that annotations based solely on accurate mass cannot confirm compound identity. According to the Metabolomics Standards Initiative (MSI) framework, such annotations are classified as putative (Level 2-3), whereas confident identification (Level 1) is reserved for matches to authentic standards using orthogonal information, including tandem MS spectra and retention time (Sumner et al., 2007; Schymanski et al., 2014).

Despite the availability of these methods, compartment-resolved metabolite baselines for field-grown legumes remain scarce, and none have been reported for soybean on the calcareous Vertisols of central and western India, where phosphorus, zinc, and iron availability are the principal constraints. This study aimed to describe and compare metabolite profiles across four compartments in a field-grown soybean system: non-rhizospheric bulk soil (NRBS), rhizospheric soil (RS), surface-sterilised roots (SRS), and unsterilised roots (SRU). This was achieved using untargeted LC-QToF-MS. This study aimed to identify compartment-associated features that could serve as candidates for future targeted investigations. This research is exploratory and hypothesis-generating, aiming to establish a metabolite baseline across the root-soil continuum and identify candidate compounds pertinent to nutrient mobilisation, root-microbe signalling, and iron acquisition, rather than elucidate mechanisms. Given that all annotations are putative and the study design incorporates three biological replicates per compartment, the data were interpreted conservatively. This study also outlines the targeted experiments necessary to test the proposed hypotheses.

## 2 Materials and methods

### 2.1 Study site and sampling

Non-rhizospheric bulk soil (NRBS) and rhizospheric soil (RS) samples were collected from a soybean field in Anta Tehsil, Baran District, within the Kota Division of southeastern Rajasthan, India (25°09′19.70″N, 76°18′39.56″E; 256 m above mean sea level). The site lies on the Hadoti (Hadauti) plateau, the northern extension of the Malwa Plateau, characterised by Deccan Trap basaltic uplands with black (Vertisol) soils (Bhattacharyya et al., 2013), which are highly suitable for soybean cultivation (Deckers et al., 2001). Sampling was conducted in August 2025 from fields planted with the soybean cultivar JS 2098 at the vegetative V7 growth stage.

Baseline fertility, based on bulk soil (NRBS) samples, showed a slightly alkaline pH of 7.29. Organic carbon content was low at 0.73%, corresponding to approximately 1.26% organic matter. The site had high levels of available phosphorus and potassium (96.7 kg ha^-1^ and 531 kg ha^-1^, respectively). Zinc, copper, iron, and manganese were available in adequate amounts, whereas exchangeable aluminium was negligible at 0.014 mg kg^-1^, which is typical of a near-neutral calcareous Vertisol (**Table 1**).

**Table 1:** Physicochemical properties and nutrient availability of pre-sowing soil, non-rhizospheric bulk soil (NRBS) and rhizospheric soil (RS).

Parameter	Pre-sowing soil	NRBS	RS
pH (1:2)	7.35 ± 0.12	7.29 ± 0.15	7.50 ± 0.15
EC (1:2) (dS m^−^¹)	0.13 ± 0.005	0.11 ± 0.002	0.09 ± 0.004
Organic C (%)	0.78 ± 0.04	0.73 ± 0.03	0.60 ± 0.02
Organic matter (%; organic C × 1.724)	1.34 ± 0.07	1.26 ± 0.05	1.03 ± 0.03
Available N (kg ha^-1^)	242 ± 3.50	230 ± 2.49	209 ± 8.85
Available P (kg ha^-1^)	104.5 ± 5.10	96.7 ± 4.27	26.5 ± 0.89
Available K (kg ha^-1^)	565 ± 8.40	531 ± 7.18	207 ± 5.22
Available Zn (mg kg^-1^)	3.25 ± 0.15	3.00 ± 0.13	0.84 ± 0.02
Available Cu (mg kg^-1^)	0.95 ± 0.02	0.89 ± 0.01	0.65 ± 0.02
Available Fe (mg kg^-1^)	6.20 ± 0.08	5.94 ± 0.03	3.64 ± 0.14
Available Mn (mg kg^-1^)	5.55 ± 0.20	5.20 ± 0.16	1.64 ± 0.04
Exchangeable Al (mg kg^-1^)	0.011 ± 0.002	0.014 ± 0.001	0.21 ± 0.008

Values are mean ± standard deviation of three biological replicates.

Pre-sowing samples were taken from the same field before sowing; NRBS and RS were collected at the vegetative V7 growth stage.

No inferential test was applied to these data (Section 3.1).

Information on field management was obtained from the growers. The previous crop at the site was garlic, and soybean was sown between late June and early July of 2025. Sampling was conducted during the vegetative V7 stage in August 2025. Mineral nutrients were applied at rates of 25 kg N, 60 kg P_2_O_5_, 40 kg K_2_O, and 20 kg S per hectare, using urea, single superphosphate, and muriate of potash, along with zinc sulphate at 5 kg per hectare as a basal micronutrient amendment. All fertilisers were applied as a basal dose at sowing via drill-sole placement. No farmyard manure, compost, vermicompost, or other organic manure was applied, nor were lime, gypsum, or micronutrient amendments, aside from zinc sulphate. Seeds were inoculated with *Bradyrhizobium japonicum* and phosphate-solubilising bacteria (PSB) prior to sowing. Representative soil samples were collected from the field before sowing to establish a pre-sowing physicochemical baseline for the soil. These samples were analysed using the procedures described below (**Table 1**). These details are provided because fertilisation history, amendment practices, and seed inoculation strongly influence root exudation profiles and rhizosphere microbial community composition, which are crucial for interpreting the soil-chemical and metabolomic differences discussed below. Section 4.4 addresses the implications of inoculation for the root compartment comparison.

Bulk soil was collected from inter-row spaces to avoid the influence of individual root systems, whereas rhizosphere soil was obtained as the soil closely adhering to roots after gently removing the loosely bound soil. All samples were collected in biological triplicates and placed in sterile bags. Each of the three biological replicates per compartment represented an independent field-sampling unit rather than a technical replicate or repeated measurement of the same unit. Specifically, NRBS and RS replicates were collected from distinct sampling locations within the field, and SRS and SRU replicates were obtained from separate root systems. Thus, n = 3 indicates three independent biological field samples per compartment drawn from a single field, thereby representing within-field biological variation. Given that untargeted metabolomics captures a labile pool of small molecules that rapidly turn over, samples intended for metabolite profiling were not dried. Heat and air drying can result in the loss of volatile and thermolabile metabolites, and during slow water removal, residual enzymatic activity, such as lipoxygenase-mediated oxylipin and jasmonate formation and glycoside hydrolysis, can produce post-sampling artifacts (Swenson et al., 2015). Consequently, the samples were transported under cold conditions, stored directly at -20 °C, and processed for metabolite extraction the following day. This brief interval between freezing and extraction was designed to minimise the freeze-associated degradation (Dunn et al., 2011). The root material stored at -20 °C was assigned to its respective treatment (surface-sterilised, SRS, or washed but non-sterilised, SRU; Section 2.2) immediately before extraction.

A distinct subsample of each soil, reserved during sampling for physicochemical characterisation, was air-dried at ambient temperature and sieved through a 2-mm mesh before analysis using established methods. The pH and electrical conductivity (EC) were determined potentiometrically in a 1:2 (w/v) soil-to-water suspension. The organic carbon content was measured using the Walkley-Black wet oxidation method (Walkley and Black, 1934). Available nitrogen was evaluated using the alkaline permanganate method (Subbiah and Asija, 1956), and available phosphorus was quantified using the Olsen method (Olsen et al., 1954). Available potassium was assessed through flame photometry (Hanway and Heidel, 1952), and available Zn, Cu, Fe, and Mn were determined by DTPA extraction, followed by atomic absorption spectrophotometry (Lindsay and Norvell, 1978). Plant-available (exchangeable) Al was extracted using unbuffered 1 M KCl and quantified via inductively coupled plasma optical emission spectrometry (ICP-OES) (Bertsch and Bloom, 1996). All physicochemical analyses were conducted in biological triplicates, with results reported as mean ± standard deviation. These procedures are classical reference methods underpinning the fertility-class norms for Indian Vertisols and are prescribed in current soil analysis manuals (Estefan et al., 2013; Stott, 2019; Sparks et al., 2020; Westville Academic Division, 2021). These methods were retained to ensure that the reported values were directly comparable with the extensive published literature on these soils. Specifically, retaining the Walkley-Black procedure maintains comparability with long-term datasets, where changes in analytical methods can introduce discontinuities that outweigh the modest accuracy gains offered by dry combustion (Grahmann et al., 2023). In discussing the differences between NRBS and RS, we focused on the most substantial and consistent differences, interpreting minor differences with caution, given the triplicate design.

### 2.2 Root sampling and surface sterilisation

The root systems of soybean plants (cultivar JS 2098; vegetative V7 stage) were excavated, placed in sterile bags, and stored at -20 °C, as outlined in Section 2.1. Prior to extraction, the root material was thawed and assigned to one of two treatments, defining the root compartments under comparison: surface-sterilised roots (SRS) and washed but non-sterilised roots (SRU). For SRS treatment, roots were washed under running tap water to remove adhering soil, then surface-sterilised by sequential immersion in 70% (v/v) ethanol for 1 min, sodium hypochlorite (4% available chlorine) for 3 min, and 70% ethanol for 30 s, followed by five to six rinses in sterile distilled water. The efficacy of sterilisation was verified by plating aliquots of the final rinse water on 10% tryptic soy agar supplemented with 50 mg L^-1^ of the antifungal imazalil and incubating at 28 °C for 2–15 days; the absence of colonies indicated effective removal of culturable surface microorganisms (Kuklinsky-Sobral et al., 2004). In contrast, SRU roots were washed to remove bulk soil but were not surface-sterilised, thus retaining the closely root-associated (rhizoplane/endophytic) microbial community. It should be noted that while sterilisation removes culturable surface microorganisms, it does not confirm axenic tissue, a consideration addressed in the interpretation (Section 4).

### 2.3 Metabolite extraction and LC-QToF-MS analysis

Approximately 1 g (fresh weight) of each soil sample or treated root material (SRS or SRU; see Section 2.2), previously stored at -20 °C, was thawed and weighed into 2.0 mL tubes for each replicate. To these tubes, 1000 µL of pre-cooled methanol:chloroform:water (2:2:1, v/v/v) at -20 °C was added directly to inhibit enzymatic activity during thawing (Saw et al., 2021). The homogenates were vortexed and sonicated in an ice-water bath for 15 min, incubated at -20 °C for 60 min, and centrifuged at 14,000 × g for 20 min at 4 °C (Zenati et al., 2023). The resulting supernatants were vacuum-dried, reconstituted in 100 µL of a 1:1 (v/v) solvent, centrifuged again, and filtered through 0.22-µm PTFE membranes before analysis. Pooled quality control (QC) samples were prepared by combining equal aliquots of all extracts and analysed periodically throughout the run to ensure analytical stability. Extraction blanks were processed concurrently with the samples (Dunn et al., 2011).

Chromatographic separation was performed using an Agilent 1260 Infinity II HPLC system coupled to an Agilent 6550 Q-ToF mass spectrometer. The separation process utilised an Agilent Eclipse XDB-C18 column (3 × 150 mm, 3.5 µm) maintained at 40 °C. The mobile phase consisted of 0.1% formic acid in water (A) and 0.1% formic acid in acetonitrile (B), with a flow rate of 0.3 mL min^-1^. The gradient was initiated at 5% B for 2 min, increased linearly to 95% B by 25 min, was maintained until 28 min, and subsequently re-equilibrated. Data acquisition was performed using a dual Agilent Jet Stream electrospray source operating across an m/z range of 100-1700. The injection volume was 5 µL. Pooled QC samples were injected every 10 samples. Data were acquired exclusively in positive electrospray ionisation mode; negative-mode data were not acquired.

### 2.4 Data processing and annotation confidence

Raw data were processed using MS-DIAL (Tsugawa et al., 2015) for peak detection, deconvolution, and alignment. Features were annotated by matching the accurate masses to spectral and compound databases, including the RIKEN Plant Metabolome MetaDatabase (Fukushima et al., 2022) and MassBank (Horai et al., 2010). The processing parameters were as follows: MS1 tolerance of 0.0001 Da, MS2 tolerance of 0.01 Da, minimum peak height of 1000, maximum charged-slice width of 0.1 Da, and sigma window of 0.5. Peak height was used to measure the relative feature abundance.

As acquisition was restricted to positive electrospray ionisation (Section 2.3), a single MS-DIAL alignment was generated. All feature detection, alignment, feature counting, differential abundance testing, and downstream interpretation reported here were derived from a single dataset; no positive- or negative-ionisation matrices were merged. The feature counts shown in **Figure 1** and the statistics reported in Section 3.5 refer to the positive-mode dataset. Restricting to a single polarity limits coverage of acidic and anionic metabolites, a constraint revisited in Section 4.7.

**Figure 1:**
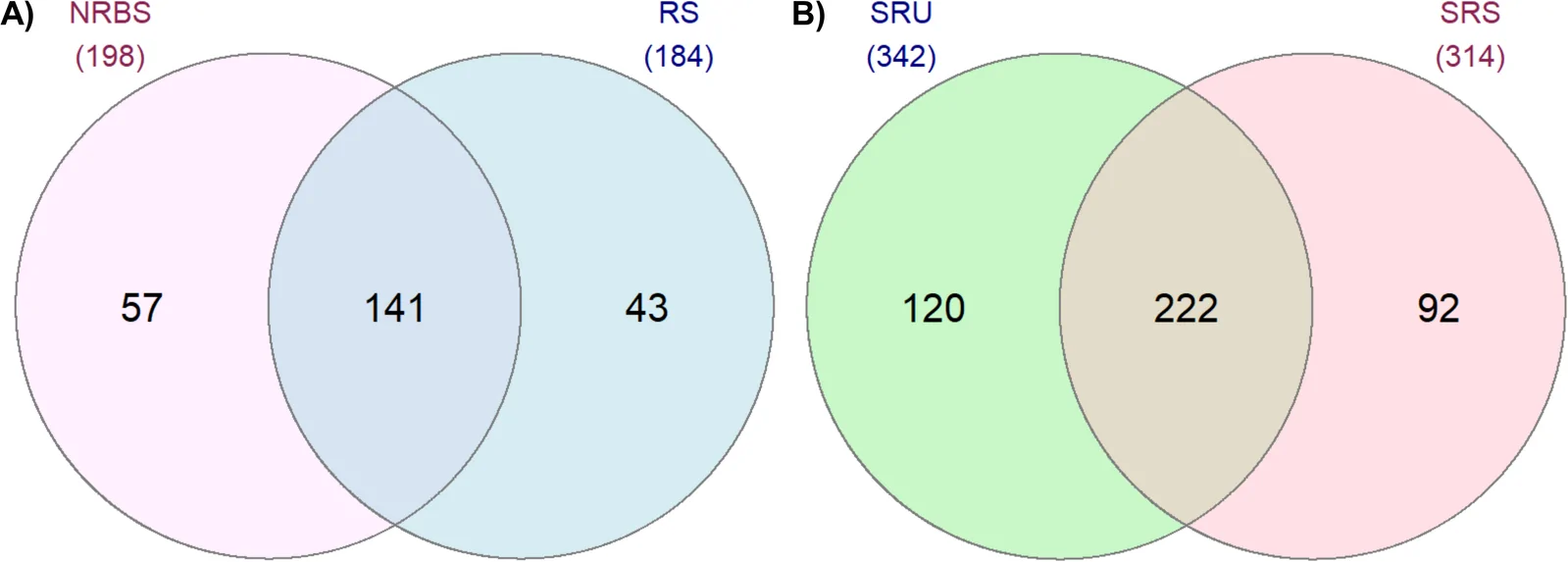
Set analysis (Venn diagrams) of detected features. **(A)** Shared and exclusive features between NRBS and RS. **(B)** Shared and exclusive features between SRU and SRS. Counts refer to the positive-ionisation feature table (Section 2.4) and are detected-feature counts, not numbers of statistical tests.

Feature identifiers were assigned independently within the soil alignment (NRBS versus RS) and root alignment (SRS versus SRU), so the same numeric identifier could represent different features in the two analyses. To prevent ambiguity, identifiers are prefixed with S_ for soil alignment and R_ for root alignment throughout the main text, **Table 2** and **Supplementary Tables S1–S4**. The MetaboLights deposit retains the original unprefixed identifiers, which differ from those used here only by the absence of the prefix.

**Table 2:** Features differing between unsterilised (SRU) and sterilised (SRS) soybean roots that met the Benjamini–Hochberg FDR threshold of adjusted *p* < 0.05, together with one near-threshold feature.

Feature ID	Putative annotation (accurate-mass match)	Putative superclass	Raw *p*-value	Adjusted *p*-value	Difference in means (log_2_FC)
R_met_88§	Benzo[a]quinolizinium alkaloid (tetrahydroisoquinoline-type)	Alkaloids and derivatives	4.409 × 10^-4^	0.0257	−10.831
R_met_34	Picene/triterpenoid dicarboxylic-acid glycoside	Lipids and lipid-like molecules	5.510 × 10^-4^	0.0257	−7.442
R_met_365†	Terfenadine (biologically implausible; likely isobaric —see Section 4.6)	Benzenoids	6.067 × 10^-4^	0.0257	−2.414
R_met_30‡	Triterpenoid (dammarane-type) glycoside acetate	Lipids and lipid-like molecules	1.657 × 10^-3^	0.0526	+8.492

All annotations are putative (MSI Levels 2–3); “Difference in means” (dm) is on the analysis scale; negative values indicate lower abundance in SRU. Benjamini–Hochberg correction was applied across the m = 127 features tested in this comparison (Section 2.5); the three features meeting the threshold share an adjusted p-value because the adjustment is monotone in rank. †Terfenadine is biologically implausible in this system and is a probable isobaric misannotation (Section 4.6). ‡R_met_30 (adjusted p = 0.0526) did not meet the significance threshold and is listed as a near-threshold candidate, not as a feature that survived correction. §The fold-change for R_met_88 derives from near- or below-detection abundances in SRU and is better read as a presence/absence contrast (Section 2.5).

As spectra were acquired and analysed at the MS1 level without tandem-MS confirmation using authentic standards, all annotations presented here are considered putative and correspond to MSI Levels 2–3 confidence (Sumner et al., 2007; Schymanski et al., 2014). In this context, compound names denote features whose accurate masses align with the named compound or its isomers, rather than confirmed identifications. Accurate-mass annotation is unable to differentiate isomers or many isobaric compounds; consequently, some database matches to compounds that are biologically implausible in an agricultural soybean system (e.g., human pharmaceuticals, hormones, and controlled substances) are likely isobaric misannotations of plant- or soil-derived isomers and are excluded from biological interpretation (refer to Section 4.6 and **Supplementary Tables S1**-**S3**).

### 2.5 Statistical analysis

Three biological replicates per compartment were used for the analyses. Preprocessing, normalisation, and differential abundance analyses were conducted in R using the MetaboDiff package (Mock et al., 2018). Features were filtered to retain only those with a coefficient of variation (CV) below 30% in the QC samples and detected in 100% of the replicates of at least one sample group. For differential-abundance testing, an additional criterion was imposed: a feature had to retain at least two valid replicate values in each of the two compartments being compared to estimate within-group variance. The implications of this second filter for the number of tests performed are detailed at the end of this section. Differential abundance between paired compartments was assessed using two-tailed independent t-tests. To mitigate false positives from multiple testing, the Benjamini-Hochberg false-discovery-rate (FDR) correction was applied, and only features meeting the pre-specified threshold of adjusted *p* < 0.05 were reported as statistically significant. Features with adjusted *p*-values slightly above this threshold are reported, where relevant, as near-threshold candidates and are not described as having survived correction. We explicitly distinguished between (i) features that passed FDR correction, (ii) features with significant fold changes that did not pass FDR correction, which are considered candidate observations requiring validation, and (iii) qualitative “compartment-exclusive” features identified in one compartment but not the other. Principal component analysis (PCA) and hierarchical clustering (based on Z-scored feature abundances) were performed in MetaboAnalyst 6.0 (Pang et al., 2024) to visualise the global structure, and variable-importance-in-projection (VIP) scores were used to rank the features contributing to compartment separation. Set relationships, including shared and exclusive features, were visualised using InteractiVenn (Heberle et al., 2015). Pathway-level patterns were investigated using the MetaboAnalyst pathway analysis module. Given that pathway mapping inherits the uncertainty of the underlying putative annotations, the pathway results are considered exploratory. Class composition and figures were compiled in R using the readxl, dplyr, tidyr, ggplot2, and patchwork packages.

Missing or undetected values were not encoded as zero. The filter required that each tested feature be detected in all replicates of at least one compartment, ensuring reliable presence in at least one compartment group. Consequently, residual undetected entries, recorded as NA, occur when a feature falls below the detection limit in the other group. These entries were imputed using the k-nearest-neighbour procedure implemented in MetaboDiff (knn_impute; missingness cutoff = 0.3), which estimates missing values based on the abundance profiles of the most similar features and samples, rather than substituting zero or an arbitrary constant. Because fold-changes are computed on imputed, log_2_-transformed abundances, analysis-scale values need not correspond exactly to ratios of representative raw peak heights. The imputed data were log_2_-transformed prior to testing, in accordance with standard practice for left-censored metabolomics data (Wei et al., 2018). Given that k-nearest-neighbour imputation relies on a limited number of neighbours (n = 3 per group), it introduces uncertainty in the affected fold-changes. Features whose fold-change is derived from abundances near or below detection in one compartment (e.g., R_met_88, log_2_FC ≈ −10. 8) are thus interpreted as qualitative presence/near-absence contrasts rather than as precisely quantified ratios. Consequently, the number of features entering the differential-abundance test is substantially smaller than the number detected. Of the 434 features detected across the two root compartments (**Figure 1B**), 222 were detected in both compartments, and of these, 127 retained at least two valid replicate values in each compartment. These 127 features constitute the tested set for the SRU versus SRS comparison, and m = 127 is the multiple-testing denominator for every adjusted *p*-value reported in Section 3.5, **Table 2**, and **Supplementary Table S4**. The counts shown in **Figure 1** represent detected-feature counts and should not be interpreted as numbers of statistical tests. Because the Benjamini-Hochberg method is a step-up procedure, adjusted *p*-values are constrained to be monotone in rank; the three features meeting the threshold therefore share the value derived from the third-ranked feature (6.067 × 10^-4^ × 127/3 = 0.0257) rather than each carrying an independently computed value. The same filtering and correction procedure was applied separately to the NRBS versus RS soil comparison.

### 2.6 Data accession

Raw and processed LC-QToF-MS data were deposited in the MetaboLights repository under the accession number MTBLS15058. The Data Availability Statement also reports this accession.

## 3 Results

### 3.1 Soil physicochemical differences between bulk and rhizospheric soil

Soil chemistry differed markedly between non-rhizospheric bulk soil (NRBS) and rhizospheric soil (RS), as detailed in **Table 1**. Prior to sowing, the soil had a pH of 7.35 ± 0.12, organic carbon of 0.78 ± 0.04%, available nitrogen of 242 ± 3.50 kg ha^-1^, available phosphorus of 104.5 ± 5.10 kg ha^-1^, available potassium of 565 ± 8.40 kg ha^-1^, available zinc of 3.25 ± 0.15 mg kg^-1^, copper of 0.95 ± 0.02 mg kg^-1^, iron of 6.20 ± 0.08 mg kg^-1^, manganese of 5.55 ± 0.20 mg kg^-1^, and exchangeable aluminium of 0.011 ± 0.002 mg kg^-1^ (**Table 1**). The bulk-soil values at the V7 sampling stage were similar to these pre-sowing values, suggesting that rhizosphere contrasts developed during the growing season rather than from pre-existing field heterogeneity. Notable differences in soil chemistry between NRBS and RS were observed (**Table 1**), with the most pronounced being marked reductions in available macro- and micronutrients in the rhizosphere. Specifically, available phosphorus in RS was 26.5 ± 0.89 kg ha^-1^, compared with 96.7 ± 4.27 kg ha^-1^ in NRBS, and available potassium was 207 ± 5.22 kg ha^-1^ in RS versus 531 ± 7.18 kg ha^-1^ in NRBS. Furthermore, available zinc and manganese were substantially lower in RS (Zn 0.84 ± 0.02 vs. 3.00 ± 0.13 mg kg^-1^; Mn 1.64 ± 0.04 vs. 5.20 ± 0.16 mg kg^-1^). Available iron and copper were moderately reduced in RS (Fe 3.64 ± 0.14 vs. 5.94 ± 0.03 mg kg^-1^; Cu 0.65 ± 0.02 vs. 0.89 ± 0.01 mg kg^-1^), and organic carbon content was slightly lower in RS (0.60 ± 0.02% vs. 0.73 ± 0.03%, corresponding to 1.03% and 1.26% organic matter, respectively). Differences in available nitrogen and electrical conductivity (EC) between the two zones were minimal, and soil pH was marginally higher in RS (7.50 ± 0.15) than in NRBS (7.29 ± 0.15). Given the overlap of the standard deviations at n = 3, the differences in pH and EC should be regarded as minor and interpreted with caution. No inferential test was conducted on the soil chemistry data; differences are described by their magnitude relative to the replicate standard deviations, as reiterated in Section 4.7, item (v).

In contrast to the nutrient elements, exchangeable aluminium was elevated in the rhizosphere, increasing by approximately 15-fold from 0.014 ± 0.001 mg kg^-1^ in NRBS to 0.21 ± 0.008 mg kg^-1^ in RS. This was the only measured element that accumulated in the root zone rather than being depleted (**Table 1**).

### 3.2 Global metabolomic separation among compartments

Untargeted LC-QToF-MS analysis identified distinct metabolite profiles across the four compartments. In the principal component analysis (PCA), the three rhizosphere-soil (RS) replicates clustered tightly and were positioned along the first component, in contrast to the more dispersed bulk-soil (NRBS) replicates (PC1 33.7%, PC2 29.4%; **Figure 2A**). Although the 95% confidence regions of the two soil compartments overlap, indicating a tendency rather than a distinct separation, the two root compartments showed complete separation along the first component, with no overlap between surface-sterilised (SRS) and unsterilised (SRU) roots (PC1 34.7%, PC2 25.3%; **Figure 2B**). The three-dimensional score plots further supported the two-dimensional separation (**Supplementary Figure S1**). The separation between root compartments was more pronounced than that between the two soil compartments, indicating that the presence or absence of the closely root-associated microbiome contributes more to profile differences than the contrast between bulk and rhizosphere soil.

**Figure 2:**
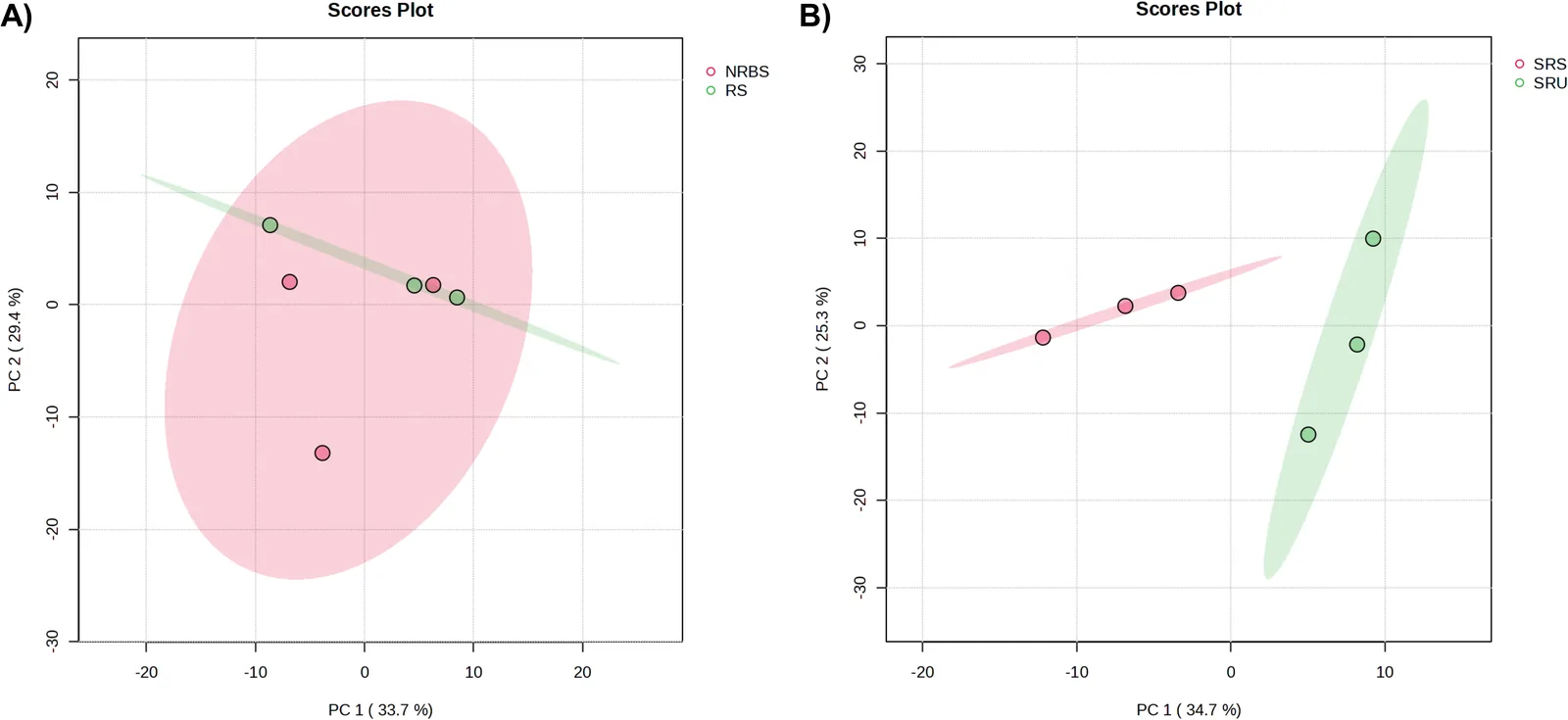
Principal component analysis (PCA) score plots of untargeted LC-QToF-MS profiles. **(A)** Non-rhizospheric bulk soil (NRBS) versus rhizospheric soil (RS). **(B)** Surface-sterilised roots (SRS) versus unsterilised roots (SRU). Percentages on the axes indicate the variance explained by each component.

### 3.3 Shared and compartment-exclusive features

The analysis quantified the overlap and exclusivity of detected features across compartments, as illustrated in **Figure 1**. In the soil comparison, 141 features were common to both NRBS and RS, while 57 were unique to NRBS and 43 were exclusive to RS (see **Figure 1A**). In the root comparison, 222 features were shared between SRS and SRU, with 120 unique to SRU and 92 exclusive to SRS (see **Figure 1B**). Hierarchical clustering of differentially abundant features (**Supplementary Figures S2**, **S4**) and VIP rankings (**Supplementary Figures S3**, **S5**) were used to summarise the distinguishing features of the soil and root pairs, respectively. Features exclusive to each compartment are reported as qualitative observations, indicating detection in one compartment but not the other under the applied processing parameters. These annotations, like all others presented here, require targeted confirmation.

### 3.4 Metabolite class composition

The chemical-class composition, as deduced from the putative annotations, varied across compartments (**Figure 3**). In bulk soil (NRBS), the predominant classes were benzene and substituted derivatives (15.47%), prenol lipids (12.71%), and carboxylic acids and derivatives (11.05%), with 17.13% of features distributed among classes each constituting less than 0.5% (**Figure 3A**). The rhizosphere soil (RS) showed a generally similar profile, with benzene and substituted derivatives at 16.07%, carboxylic acids and derivatives at 10.71%, and prenol lipids at 8.93%. However, it had a markedly higher proportion of features annotated as flavonoids (4.76% compared to 1.66% in NRBS) and a slightly higher proportion annotated as steroids and steroid derivatives (7.14% compared to 6.63%) (**Figure 3B**). The enrichment of flavonoids aligns with the RS-exclusive flavonoid features detailed in Section 3.6. Sterilised roots (SRS) were primarily composed of prenol lipids (22.97%), followed by fatty acyls (9.46%), and carboxylic acids and derivatives and steroids and steroid derivatives (7.21% each) (**Figure 3C**). Unsterilised roots (SRU) exhibited an even higher proportion of prenol lipids (25.15%) and steroids and steroid derivatives (7.89%), with flavonoids also slightly higher than in SRS (4.09% compared to 3.60%) (**Figure 3D**). It is crucial to note that these proportions reflect the composition of the annotated feature set rather than confirmed compound classes or relative abundances, as class assignment relies on the underlying putative annotations and accounts for features rather than signal.

**Figure 3:**
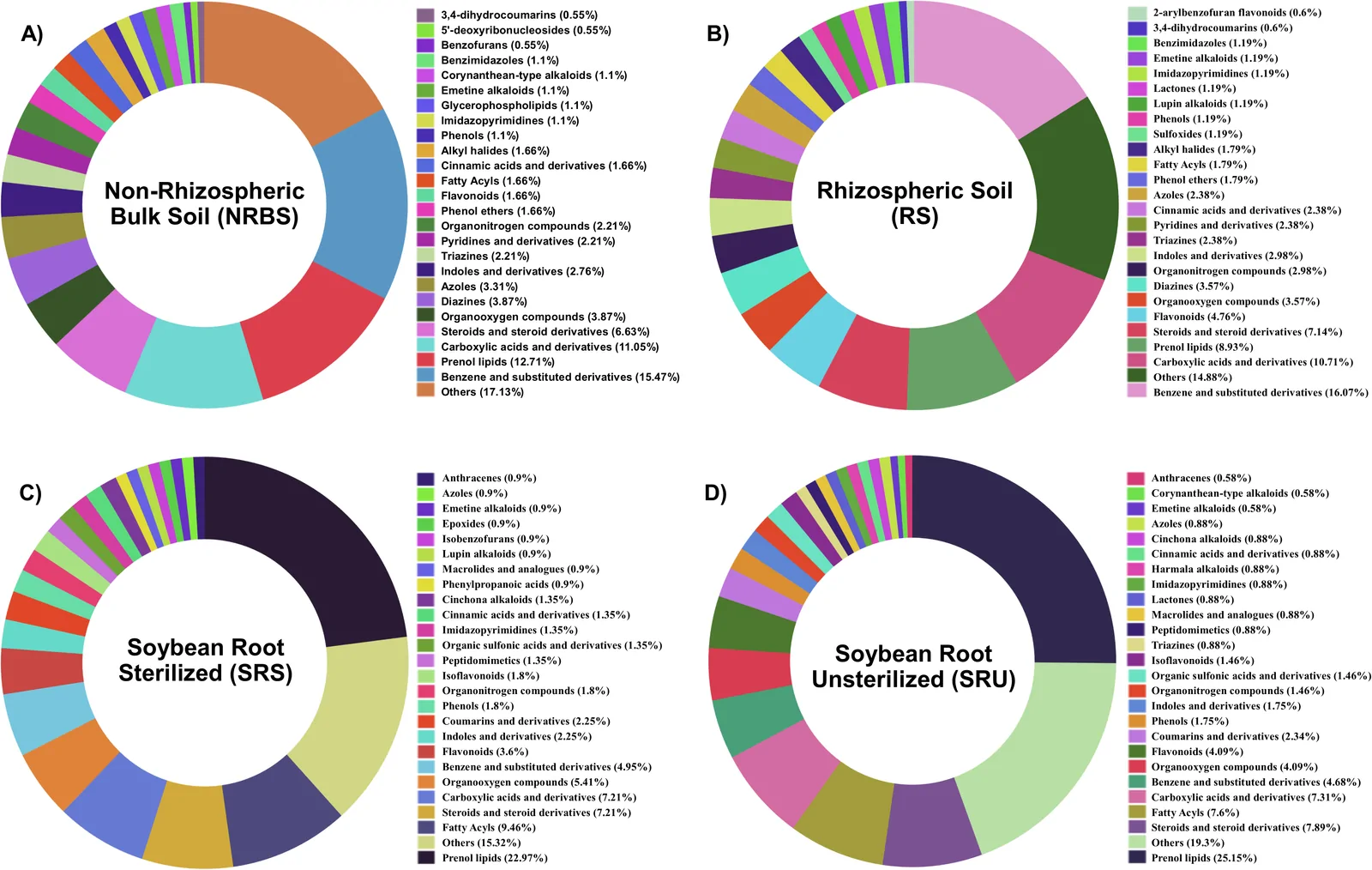
Relative proportions of putative metabolite chemical classes, based on accurate-mass annotations, in **(A)** NRBS, **(B)** RS, **(C)** SRS and **(D)** SRU. Classes are ordered by proportion, and those individually representing less than about 0.5% of annotated features are pooled as “Others”. Percentages denote the proportion of annotated features assigned to each class, not relative abundance.

### 3.5 Differential features between root compartments

To distinguish between sterilised (SRS) and unsterilised (SRU) roots, t-tests with Benjamini-Hochberg correction were conducted on 127 features that met the quality-control and variance-estimability criteria for this analysis (m = 127; Section 2.5; **Table 2**). Using the predetermined threshold of adjusted *p* < 0.05 (Section 2.5), three features remained significant after FDR correction: a benzo[a]quinolizinium alkaloid (R_met_88; raw *p* = 4.409 × 10^-4^, adjusted *p* = 0.0257), a picene/triterpenoid dicarboxylic-acid glycoside (R_met_34; raw *p* = 5.510 × 10^-4^, adjusted *p* = 0.0257), and terfenadine (R_met_365; raw *p* = 6.067 × 10^-4^, adjusted *p* = 0.0257). These features share the same adjusted *p*-value because the Benjamini-Hochberg adjustment is monotonic (Section 2.5). A fourth feature, a triterpenoid acetate (R_met_30; adjusted *p* = 0.0526), slightly exceeded the threshold and is therefore reported as a near-threshold candidate rather than a feature that survived correction. All three significant features showed lower abundance in SRU (negative difference in means), whereas the near-threshold R_met_30 was more abundant in SRU. The reported fold-change for R_met_88 (log_2_ FC ≈ −10.8) indicates abundances near or below detection limits in SRU and should be interpreted as a presence/absence contrast rather than a quantitative ratio (Section 2.5). Notably, the FDR-corrected set predominantly includes features with chemically generic annotations or, in the case of terfenadine, biologically implausible annotations within this system, suggesting potential isobaric interference (Section 4.6); thus, statistical robustness does not necessarily equate to accurate identification.

Several features of greater biological interest, which showed substantial abundance differences between root compartments, did not withstand FDR correction and are therefore reported as candidate observations rather than statistically robust differences. A feature identified as jasmonic acid (R_met_1) was markedly lower in SRU than in SRS (peak height ≈ 89 vs ≈ 9650; log_2_FC = −6.76, raw *p* = 0.025, adjusted *p* = 0.254). Its precursor, 12-oxophytodienoic acid (OPDA, R_met_69), varied in the opposite direction, being higher in SRU (log_2_ FC = +4.10, raw *p* = 0.090, adjusted *p* = 0.327); the two features therefore do not describe a coordinated shift in the jasmonate pathway, and neither difference survived correction. A feature identified as daidzein (R_met_208) was more abundant in SRU than in SRS (peak height ≈ 455000 vs ≈ 56800; log_2_ FC = +3.41, raw *p* = 0.034, adjusted *p* = 0.254), a difference that likewise did not survive correction. Features identified as core structural lipids, such as palmitic acid (R_met_337) and dihydrosphingosine (R_met_222), were more abundant in SRU than in SRS. These comparisons are based on relative peak heights from putative annotations across three biological replicates and require targeted validation (Section 4). **Supplementary Table S4** provides the full set of large-fold-change features with their raw and adjusted *p*-values, distinguishing those that met the FDR threshold from the candidate observations that did not.

### 3.6 Compartment-exclusive features of interest in the rhizosphere and colonised roots

Among the 43 soil features unique to RS, several were identified as flavonoids and related phenylpropanoids. These include features corresponding to kaempferide (S_met_206), peonidin-3,5-O-diglucoside (S_met_231), quercetin-3-(6-O-acetylglucoside) (S_met_233), and isorhamnetin-3-O-rutinoside (S_met_226). Additionally, a feature identified as an organoheterocyclic lactone, specifically delta-tridecalactone (S_met_239), was detected. **Supplementary Table S2** lists the 25 most abundant RS-exclusive features; however, S_met_233 and S_met_226 are not included in that subset. Notably, no feature annotated as a low-molecular-weight organic acid was present among the RS-exclusive features. Among the 120 root features exclusive to SRU, the most prevalent was the microbial siderophore arthrobactin (C_20_H_36_N_4_O_9_, R_met_400). A second, structurally related linear trihydroxamate (C_20_H_36_N_4_O_8_, R_met_125) was also detected among the SRU-exclusive features, and a feature annotated as citric acid (R_met_198) was likewise detected only in SRU (see **Supplementary Table S3**). These compartment-exclusive features are qualitative and putative. In particular, the annotation of the siderophore is treated with caution in Section 4.3, as many soil taxa produce structurally similar siderophores and accurate mass alone is insufficient to determine a specific structure or biological origin.

### 3.7 Pathway-level patterns

Exploratory pathway mapping of putatively annotated features revealed distinct dominant pathways across compartments (**Figure 4**). Bulk soil (NRBS) was predominantly associated with taurine/hypotaurine and arginine/proline metabolism (**Figure 4A**), whereas rhizosphere soil (RS) was primarily linked to alanine, aspartate, and glutamate metabolism (**Figure 4B**). Sterilised roots (SRS) were most strongly associated with linoleic acid metabolism (**Figure 4C**), whereas unsterilised roots (SRU) showed a shift towards isoflavonoid and flavone/flavonol biosynthesis (**Figure 4D**). Given the uncertainty inherent in the underlying putative annotations, pathway assignment is considered exploratory and hypothesis-generating rather than definitive evidence of pathway-level flux.

**Figure 4:**
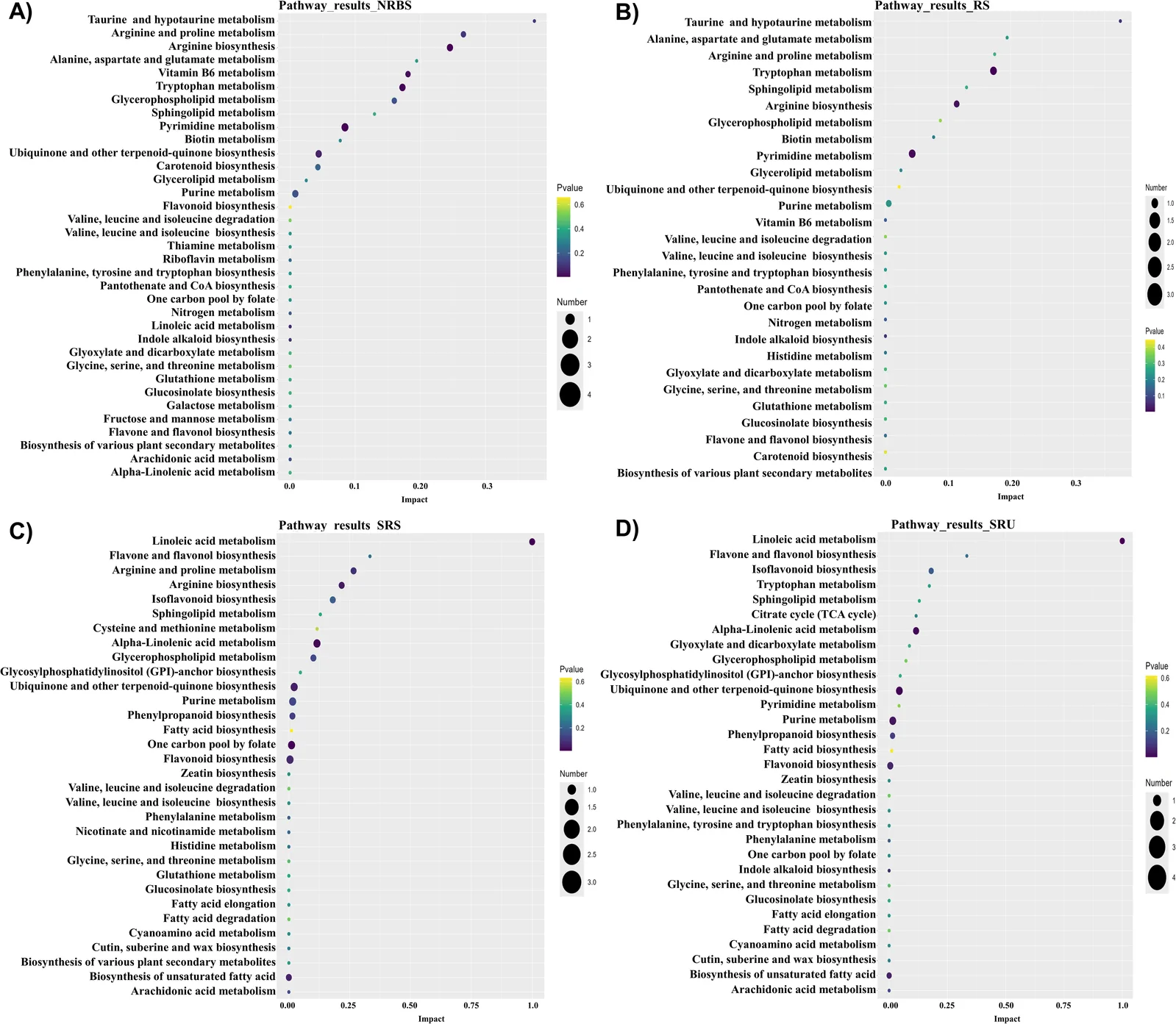
Exploratory metabolic-pathway mapping of putatively annotated features for **(A)** NRBS, **(B)** RS, **(C)** SRS and **(D)** SRU. The x-axis is pathway impact; point size reflects the number of mapped features; colour reflects the mapping *p*-value. Pathway results are exploratory (see Section 3.7).

## 4 Discussion

We conducted a comprehensive profiling of four compartments in a field-grown soybean system using untargeted LC-QToF-MS. The analysis revealed distinct separation among bulk soil, rhizosphere soil, and both sterilised and unsterilised roots, along with compartment-specific soil chemistry. In the following discussion, we interpret these findings within the framework of established rhizosphere biology. It is important to note that all metabolite annotations are provisional (MSI Level 2–3), and the differential-abundance signals of primary biological interest did not withstand multiple-testing correction. Furthermore, the study design is limited to a single site and time point, with three biological replicates. Consequently, our interpretations are presented as hypotheses for future testing, and we outline the necessary targeted experiments.

### 4.1 Rhizosphere nutrient depletion and aluminium behaviour

The soil data indicate a marked depletion of available phosphorus, potassium, zinc, and manganese in the rhizosphere relative to the bulk soil (**Table 1**). This depletion in the immediate root zone is expected due to root uptake and is a commonly reported feature of rhizosphere soil chemistry. The direction and magnitude of the differences in P, K, Zn, and Mn are substantial and consistent across replicates, making them the most reliable soil-chemistry findings of the study. In contrast, the minor differences in pH and electrical conductivity (EC) should be interpreted with caution. Although the marginal increase in rhizosphere pH (from 7.29 to 7.50) is directionally consistent with the alkalinisation that may occur with nitrate-driven anion uptake and the associated efflux of hydroxyl/bicarbonate ions in legumes (Israel and Jackson, 1982; Haynes, 1990; Rao et al., 2002), the overlapping standard deviations across three replicates prevent us from establishing this shift with confidence, and we do not construct a mechanistic argument based on it. The analysis of the phosphorus contrast must account for the phosphate-solubilising bacterial inoculum applied to the seed at this location (refer to Section 2.1). This inoculum is expected to enhance microbial phosphorus mobilisation and uptake within the root zone, potentially influencing the extent of the depletion observed in this study.

Aluminium warrants distinct consideration, as the approximately 15-fold increase in KCl-exchangeable Al (from 0.014 to 0.21 mg kg^-1^; **Table 1**) was the only element to accumulate in the root zone, representing the most consistent soil-chemical contrast in the dataset. Interpretation of this phenomenon must begin with scale: at pH levels of 7.3-7.5, absolute concentrations remain exceedingly low, several orders of magnitude below those linked with aluminium phytotoxicity in acidic soils. Thus, the result indicates mobilisation of a minor pool rather than the onset of aluminium stress. Three non-exclusive processes could account for this. Firstly, root and microbial activity chemically alter the immediate root zone, and even minor, spatially localised decreases in pH at the root surface markedly increase aluminium solubility compared to the bulk soil (Hinsinger, 2001). Secondly, and most pertinent here, soybean roots release low molecular weight carboxylates, primarily citrate and malate, in response to both aluminium exposure and phosphorus deficiency, with these anions chelating Al^3+^ in the rhizosphere (Yang et al., 2000; Dong et al., 2004; Liao et al., 2006; Kochian et al., 2015). Since unbuffered KCl extracts both organically complexed and free exchangeable aluminium (Bertsch and Bloom, 1996), carboxylate-mediated complexation is expected to shift aluminium into this operationally defined pool; thus, the observed increase aligns with carboxylate exudation rather than an increase in free, toxic Al^3+^. The concurrent 3.6-fold depletion of available phosphorus (**Table 1**) is notable in this context, as phosphorus deficiency strongly triggers carboxylate release in soybean, and the same carboxylates that complex aluminium also displace phosphate from calcium and iron bound forms (Jones, 1998; Ryan et al., 2001; Hinsinger, 2001). Consequently, the phosphorus and aluminium patterns may represent two outcomes of a single exudation response (Dong et al., 2004; Liao et al., 2006). Third, root-induced weathering of the smectitic clays that dominate these Vertisols could directly release clay-edge and interlayer aluminium. Aluminium at low concentrations is increasingly recognised for its beneficial as well as toxic effects on higher plants, and the concentrations recorded here fall within that low range (Bojórquez-Quintal et al., 2017; Ofoe et al., 2023).

The untargeted data presented here do not explicitly demonstrate rhizosphere carboxylate accumulation. Notably, no feature annotated as a low-molecular-weight organic acid was identified among the RS-exclusive features (Section 3.6). The only carboxylate annotated in the dataset, citric acid (R_met_198), was exclusive to unsterilised roots rather than rhizosphere soil. This limitation is partly analytical: small, highly polar organic anions are poorly retained on the reversed-phase column used and ionise weakly under the acidified acquisition conditions. Thus, their absence from the feature table does not confirm their absence from the soil (Section 4.7). Furthermore, the current data cannot differentiate among the three proposed mechanisms, as exchangeable aluminium was measured at a single time point using a single operationally defined extraction. Consequently, we propose carboxylate exudation as the mechanistic hypothesis most supported by the published soybean literature and the direction of our soil-chemical data, rather than as an observation of this study. Testing this hypothesis would necessitate targeted, standard-based quantification of citrate, malate, and oxalate in rhizosphere soil solution, along with aluminium speciation.

### 4.2 A pattern consistent with rhizosphere priming

The simultaneous reduction of organic carbon and various available nutrients in the rhizosphere (**Table 1**) aligns with the rhizosphere priming effect. This effect, driven by living-root activity and rhizodeposition, modifies the turnover of soil organic matter and the mobilisation of nutrients (Kuzyakov, 2010; Huo et al., 2017; Zhu et al., 2018). The exploratory pathway mapping, which revealed a transition from taurine/arginine-associated pathways in bulk soil to alanine/aspartate/glutamate-associated pathways in the rhizosphere (**Figures 4A, B**), suggests altered nitrogen turnover at the root interface (Yin et al., 2018). However, pathway mapping here relies on putative annotations and does not demonstrate flux. While the priming interpretation is consistent with the soil-chemistry data, the data do not directly support it. Testing this interpretation would require process-level measurements, such as isotopically resolved carbon turnover and extracellular-enzyme assays, which were beyond the scope of this profiling study.

### 4.3 Iron availability and a putative siderophore signature

Available iron concentrations were lower in the rhizosphere than in bulk soil (**Table 1**). Iron acquisition is a well-known challenge in aerobic, near-neutral to alkaline soils, where Fe(III) has limited solubility. Both plants and microorganisms employ iron-acquisition strategies, including the use of microbial siderophores (Jin et al., 2006; Ahmed and Holmström, 2014; Fukushima et al., 2014; Lurthy et al., 2021). Among features identified exclusively in unsterilised roots, the most prevalent was annotated as the siderophore arthrobactin (R_met_400); a second SRU-exclusive feature (R_met_125, C_20_H_36_N_4_O_8_) was annotated as a structurally related linear trihydroxamate of the arthrobactin/deferoxamine family and was the next most abundant SRU-exclusive feature, so that the two most abundant features unique to colonised roots are both consistent with hydroxamate-type siderophores. This finding aligns with the general expectation that microbial iron-acquisition compounds are associated with colonised roots, and siderophore production is known to contribute to both iron supply and competition among rhizosphere microorganisms (Crowley and Kraemer, 2007; Gu et al., 2020). However, this observation requires cautious interpretation: the annotation is tentative and based solely on accurate mass; numerous soil taxa produce structurally related hydroxamate siderophores (Boiteau et al., 2019), precluding inference of a specific structure or producing organism from these data; and the feature is reported as compartment-exclusive rather than as a validated quantitative difference. Consequently, we present the siderophore signature as a candidate observation that warrants targeted investigation, specifically MS/MS confirmation against authentic siderophore standards and characterisation of the root-associated microbial community, rather than as definitive evidence of a specific iron-acquisition mechanism.

### 4.4 Features associated with defence and symbiotic signalling

In examining legume-microbe interactions, two aspects of the root-compartment comparison merit consideration, both requiring explicit statistical validation. Firstly, a feature identified as jasmonic acid (R_met_1) was markedly lower in unsterilised roots than in sterilised ones, while its precursor, OPDA (R_met_69), varied in the opposite direction, being higher in unsterilised roots (Section 3.5); the pair therefore does not describe a coordinated shift in the jasmonate pathway. Transient modulation of jasmonate and related defence signalling is linked to the establishment of beneficial root symbioses in legumes and other plants (Gutjahr and Paszkowski, 2009; Pieterse et al., 2012; Aragón et al., 2026). Consequently, the reduced presence of a jasmonate-annotated feature in colonised roots aligns with existing literature. Secondly, a feature identified as daidzein (R_met_208), an isoflavone that acts as a signal in the soybean-*Bradyrhizobium* symbiosis (Kosslak et al., 1987; Graham, 1991; Schultze and Kondorosi, 1998; Subramanian et al., 2015; Sugiyama, 2019), was detected at higher concentrations in unsterilised roots. Additionally, RS-exclusive soil features included several annotated as flavonoids (Section 3.6). This finding aligns with the established role of isoflavonoids in rhizobial signalling. However, two design features must be considered in light of both observations. First, seeds were inoculated with *B. japonicum* and phosphate-solubilising bacteria prior to sowing (Section 2.1), indicating that the root-associated community sampled was augmented rather than entirely native; thus, an isoflavonoid signal in colonised roots is expected, but the comparison does not assess spontaneous colonisation. Second, and more significantly, the SRS/SRU contrast does not represent a straightforward colonised versus uncolonised comparison, as surface sterilisation constitutes a chemical treatment: sequential exposure to 70% ethanol and hypochlorite at 4% available chlorine (Section 2.2) extracts surface- and apoplast-associated metabolites and oxidises susceptible ones. An approximately eightfold reduction in the daidzein signal in sterilised roots is therefore as consistent with treatment-associated loss as with colonisation-associated accumulation; the jasmonate difference may be similarly influenced, although it runs counter to the daidzein contrast and is less easily attributed to oxidation alone. Distinguishing these possibilities requires a water-washed-only control and a mock sterilisation applied to axenically raised roots.

Neither the reduction in jasmonate levels nor the increase in daidzein levels remained significant after FDR correction (daidzein, R_met_208, had a raw *p*-value of 0.034; see Section 3.5). Both findings are based on single, putatively annotated features quantified by relative peak height in three replicates. Furthermore, the observed increase in daidzein in colonised roots compared with sterilised roots is merely one of several potential outcomes, given the numerous variables distinguishing these compartments. These findings should be considered candidate signals consistent with existing biological knowledge and warrant further investigation through targeted testing. This would involve MS/MS-confirmed, standard-based quantification of jasmonic acid, OPDA, and specific isoflavonoids across independent replicates and time points. These observations do not support the conclusion that colonisation induced these changes. We have intentionally refrained from characterising the jasmonate pattern as “immunosuppression” or the isoflavonoid pattern as definitive symbiotic signalling, as the data do not substantiate causal claims of such magnitude.

### 4.5 Structural-lipid features in colonised roots

Structural lipids, such as palmitic acid (R_met_337) and dihydrosphingosine (R_met_222), were found in higher quantities in unsterilised roots than in sterilised ones (Section 3.5). Membrane remodelling is associated with root symbiont integration, and the increased presence of structural lipids in colonised roots aligns with this expectation. As with the signalling features, these annotations are provisional and have not been confirmed by standard methods. The observed differences between compartments are attributed to both colonisation and incidental variation among root samples. To determine whether colonisation alters specific membrane lipids, a targeted lipidomic analysis would be required.

### 4.6 Bulk-soil features, apparent xenobiotics, and the problem of isobaric misannotation

A subset of features was identified exclusively in bulk soil, with putative annotations indicating compounds linked to legacy agrochemicals, such as triazine and diazine herbicides and strobilurin fungicides. The apparent confinement of these features to bulk soil, coupled with their absence in the rhizosphere, may reflect rhizosphere processes. These processes could include enhanced co-metabolic degradation, sorption to organic matter, plant uptake, or analytical ion suppression due to the dense matrix of root-derived compounds (Chaudhry et al., 2005; Kuiper et al., 2001). It is crucial to avoid interpreting the absence of an MS1 feature as definitive evidence of compound degradation, as the absence of a peak does not confirm structural breakdown. Any of the aforementioned alternative explanations could yield the same observation. Consequently, we propose rhizosphere attenuation of agrochemical residues merely as a hypothesis for future targeted investigations, which should employ authentic standards, quantitative recovery controls, and, ideally, isotope-labelled tracers.

The annotations identified as unique to bulk soil, along with other features, correspond to human pharmaceuticals, steroids, and controlled substances, including opiates, antidepressants, antivirals, and hormones (see **Supplementary Tables S1**–**S3**, in which such entries carry a footnote identifying them as probable isobaric misannotations). These annotations are biologically implausible in a rainfed agricultural soybean system and likely result from isobaric misannotations. Such misannotations occur when accurate-mass matches align with database entries that share a molecular formula or a similar mass with plant- or soil-derived isomers not included in, or incorrectly prioritised by, the reference libraries. Consequently, these annotations have been excluded from biological interpretation and are retained in the supplementary tables solely for transparency. Because these annotations cannot be distinguished from plausible ones by inspection, they are flagged wherever they appear: implausible annotations are marked with a dagger (†) in **Figure 5** and in **Table 2**. No feature has been removed from any figure or table on the grounds of implausibility; thus, the flagging is additive and conceals nothing. The corresponding entries in **Supplementary Tables S1**-**S3** carry a footnote to the same effect and should be read in conjunction with this section. Notably, one such annotation, terfenadine (R_met_365), persisted after FDR correction (see Section 3.5), highlighting a critical consideration for untargeted MS1 studies: statistical significance indicates a reproducible difference in a feature’s abundance but does not confirm correct feature identification. This underscores the necessity for MS/MS-based confirmation before considering any annotated compound from this dataset as authentic.

**Figure 5:**
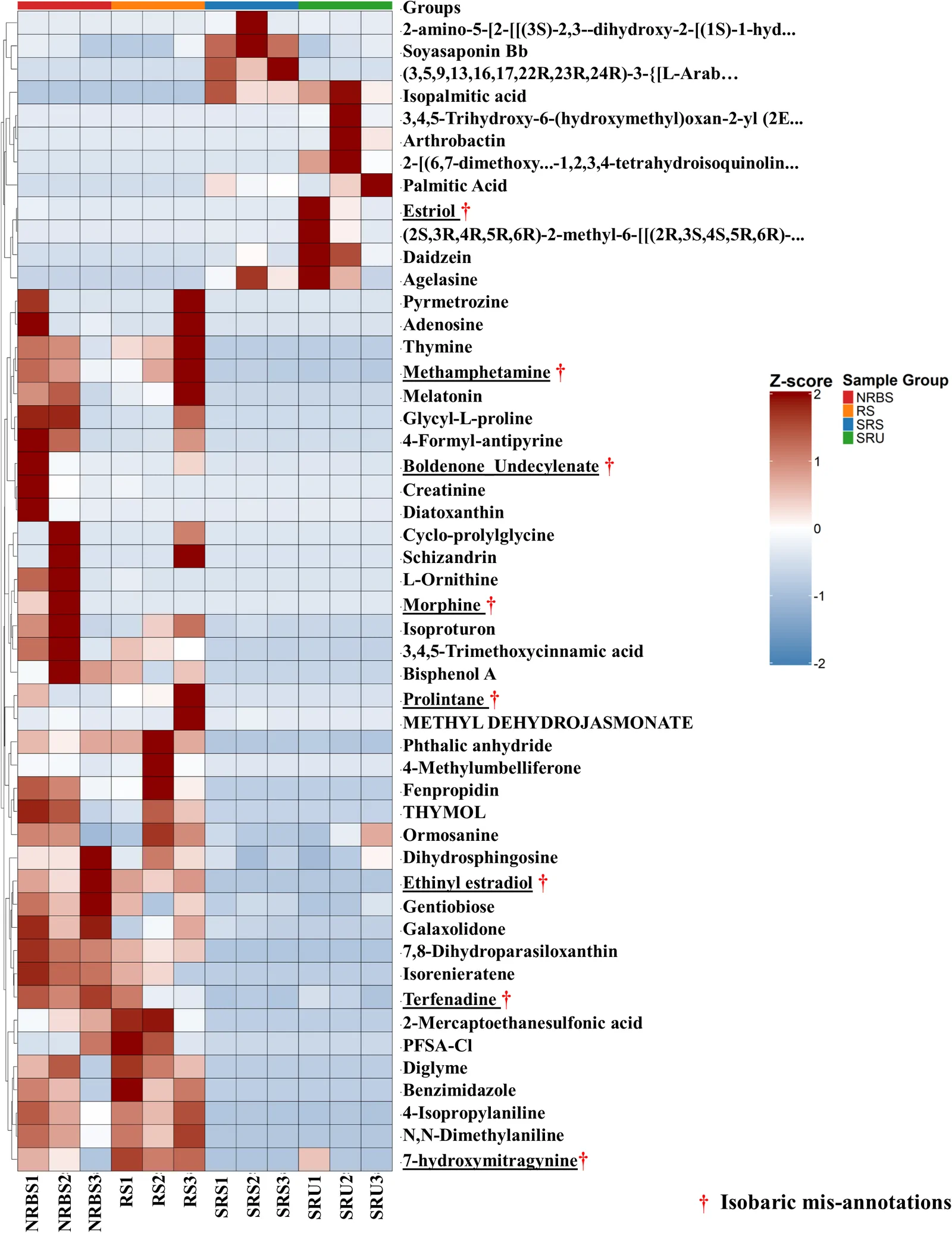
Hierarchical clustering heatmap of differentially abundant features across all biological replicates (columns: NRBS, RS, SRS, SRU; rows: features). Colour indicates the Z-score of relative feature abundance. †Annotations matching human pharmaceuticals, hormones or controlled substances are probable isobaric misannotations of plant- or soil-derived isomers and are excluded from biological interpretation (Section 4.6).

### 4.7 Limitations

This study is constrained by several limitations that affect its conclusions. (i) Annotation confidence: The data were acquired and analysed at the MS1 level without tandem-MS confirmation against authentic standards, rendering all annotations putative (MSI Level 2–3). This approach cannot resolve isomers or many isobars and, as demonstrated in Section 4.6, may yield biologically implausible annotations. (ii) Replication and design: The study employed three biological replicates from a single field site and a single sampling time. The differential abundance signals of greatest biological interest did not withstand FDR correction, necessitating independent replication across sites and phenological stages. (iii) Relative, untargeted quantification: Feature abundances are expressed as relative peak heights rather than absolute concentrations and were not calibrated against standards. Matrix effects vary among the distinct sample types (soil versus root), complicating cross-compartment comparisons. (iv) Absence of microbiome characterisation: The contrast between sterilised and unsterilised samples serves as a proxy for the presence of the closely root-associated microbiome, yet the community was not characterised. Consequently, inferences regarding microbial sources of specific features (e.g., siderophores) remain indirect. Surface sterilisation removes culturable surface microorganisms but does not ensure axenic tissue. (v) Soil-chemistry testing: Physicochemical values are averages of triplicates, and formal significance testing across all parameters was limited. Small differences (e.g., pH, EC) lack robustness at this level of replication. (vi) Pathway inference: Pathway mapping propagates annotation uncertainty and does not measure flux. (vii) Analytical coverage: The reversed-phase, formic-acid workflow used here under-represents small, highly polar, and anionic metabolites, including the low-molecular-weight carboxylates most relevant to the aluminium and phosphorus results (Section 4.1); a complementary hydrophilic-interaction or ion-chromatography separation in negative-ionisation mode would be required to cover this chemical space. (viii) Surface sterilisation as a chemical treatment: The SRS/SRU contrast confounds the presence of the root-associated microbiome with the effect of the sterilisation procedure itself, which extracts and oxidises surface-associated metabolites (Section 4.4); differences between these compartments therefore cannot be attributed to colonisation alone. (ix) Seed inoculation: The crop was raised from seed inoculated with *B. japonicum* and phosphate-solubilising bacteria (Section 2.1), so the findings describe an inoculated production system rather than an uninoculated one. (x) Single ionisation polarity: Acquisition was restricted to positive mode (Section 2.3), further limiting coverage of acidic and anionic metabolites beyond the chromatographic constraint noted in item (vii). (xi) Operational definition of rhizosphere soil: Soil adhering to roots is preferentially the fine, clay- and colloid-rich fraction, which differs from bulk soil in surface area and mineralogy independently of any root process and could contribute both to the apparent micronutrient depletion and to the elevated KCl-extractable aluminium. Collectively, these limitations suggest that the study should be interpreted as an exploratory, hypothesis-generating survey.

### 4.8 Implications and future directions

Within these parameters, the dataset provides a compartment-resolved metabolite baseline for a field-grown soybean system on a Hadoti-plateau Vertisol, identifying specific, testable candidates: root- and microbially derived carboxylates related to aluminium mobilisation and phosphorus availability, which the present reversed-phase workflow could not resolve and which require a complementary polar-metabolite method (Section 4.1); a microbial siderophore signature linked to iron acquisition (Section 4.3); and jasmonate and isoflavonoid features relevant to symbiosis establishment (Section 4.4). The subsequent logical steps include (a) targeted, MS/MS-confirmed, standard-based quantification of these candidate compounds; (b) paired characterisation of the root-associated microbial community (e.g., 16S rRNA and ITS amplicon sequencing) on the same samples to link chemistry to community; and (c) independent replication across sites, cultivars, and growth stages. Only after such validation would the specific compounds identified here be suitable candidates for exudate-based biostimulant development; we propose the biostimulant framework as a motivation for future research rather than as a conclusion of the current study.

## 5 Conclusion

Untargeted LC-QToF-MS effectively differentiated the metabolite profiles of bulk soil, rhizosphere soil, and sterilised and unsterilised soybean roots, revealing compartment-specific variations in soil chemistry. Notably, the rhizosphere showed marked depletion of available phosphorus, potassium, zinc, and manganese, alongside increased exchangeable aluminium. Root colonisation correlated with potential changes in features annotated as jasmonic acid, isoflavonoids (including daidzein), and a microbial siderophore. Although these changes align with established rhizosphere biology, they did not withstand multiple-testing correction and are based on provisional (MSI Level 2-3) annotations; of the root-compartment contrasts, only three features met the pre-specified FDR threshold, and their annotations are chemically generic or implausible. The rhizosphere aluminium increase is interpreted as the mobilisation of a small pool, plausibly through carboxylate complexation, but this mechanism is a hypothesis drawn from the soybean literature rather than an observation of the present dataset. Consequently, this study should be viewed as an exploratory, hypothesis-generating survey that establishes a metabolite baseline and identifies a set of prioritised candidate features for the soybean root-soil continuum. Verification of the identity, quantity, and biological function of these candidates requires targeted MS/MS-based analysis, microbiome characterisation, and independent replication, which we propose as essential subsequent steps before drawing any mechanistic or applied conclusions. The root-compartment signals, in particular, should be regarded as provisional for another reason: the comparison between sterilised and unsterilised roots conflates microbial colonisation with the chemical effects of the sterilisation procedure and cannot be resolved without an appropriate mock-sterilisation control.

## Data Availability

The datasets presented in this study can be found in online repositories. The names of the repository/repositories and accession number(s) can be found in the article/**Supplementary Material**.
