# Supplementary material for: Untargeted LC-QToF-MS profiling of the soybean (Glycine max) root-soil continuum reveals spatial compartmentalisation of putative exudate, defence, and iron-acquisition metabolite signatures
Source: Front Plant Sci. 2026 Sep 21;17:1940810. doi: 10.3389/fpls.2026.1940810 (PMC13635369; doi:10.3389/fpls.2026.1940810)
Supplement: Supplementary file 1 [file DataSheet1.docx]

**Supplementary Material**

**Untargeted LC-QToF-MS profiling of the soybean (*Glycine max*) root–soil continuum reveals spatial compartmentalisation of putative exudate, defence, and iron-acquisition metabolite signatures**

Kirti Suman, Hirudhaya Ravi, Pushpendra Sharma, Mahaveer P. Sharma, Minakshi Grover, Rajeev Kaushik*

**Supplementary Table S1.** Non-rhizospheric bulk soil (NRBS)-Top 25 exclusive features, with putative annotations.^1^

| **Metabolite ID** | **Metabolite Nomenclature** | **Molecular formula** | **Metabolite Superclass** | **NRBS Relative Abundance** |
| --- | --- | --- | --- | --- |
| S_met_104 | Morphine | C_17_H_19_NO_3_ | Alkaloids and derivatives | 384885 |
| S_met_161 | delta-Tocotrienol | C_27_H_40_O_2_ | Lipids and lipid-like molecules | 182741 |
| S_met_160 | beta-Tocotrienol | C_28_H_42_O_2_ | Lipids and lipid-like molecules | 76677 |
| S_met_32 | Atrazine-2-hydroxy | C_8_H_15_N_5_O | Organoheterocyclic compounds | 67703 |
| S_met_5 | 1-palmitoyl-2-oleoyl-sn-glycero-3-phosphocholine | C_42_H_82_NO_8_P | Lipids and lipid-like molecules | 63078 |
| S_met_165 | 7,8,7',8'-Tetradehydroastaxanthin | C_40_H_48_O_4_ | Lipids and lipid-like molecules | 56901 |
| S_met_82 | Hydroxybutorphanol | C_21_H_29_NO_3_ | Benzenoids | 53297 |
| S_met_158 | Vitamin K1 | C_31_H_46_O_2_ | Lipids and lipid-like molecules | 47906 |
| S_met_164 | 4-Amino-2-chloropyridine | C_5_H_5_ClN_2_ | Organoheterocyclic compounds | 47363 |
| S_met_67 | Estriol | C_18_H_24_O_3_ | Lipids and lipid-like molecules | 46975 |
| S_met_27 | Artemisinin | C_15_H_22_O_5_ | Lipids and lipid-like molecules | 46339 |
| S_met_196 | Prednisolone | C_21_H_28_O_5_ | Lipids and lipid-like molecules | 46178 |
| S_met_72 | Finasteride | C_23_H_36_N_2_O_2_ | Lipids and lipid-like molecules | 44718 |
| S_met_45 | Chloridazon | C_10_H_8_ClN_3_O | Organoheterocyclic compounds | 44116 |
| S_met_159 | alpha-Carotene | C_40_H_56_ | Lipids and lipid-like molecules | 43981 |
| S_met_170 | Capecitabine | C_15_H_22_FN_3_O_6_ | Nucleosides, nucleotides, and analogues | 43255 |
| S_met_183 | Thiamine | C_12_H_17_N_4_OS^+^ | Organoheterocyclic compounds | 42577 |
| S_met_34 | Azoxystrobin | C_22_H_17_N_3_O_5_ | Benzenoids | 40971 |
| S_met_117 | PFSA-unsaturated | - | - | 40752 |
| S_met_132 | Pyrrhoxanthinol | C_37_H_46_O_5_ | Lipids and lipid-like molecules | 38287 |
| S_met_51 | Cytidine | C_9_H_13_N_3_O_5_ | Nucleosides, nucleotides, and analogues | 36877 |
| S_met_110 | Orotic acid | C_5_H_4_N_2_O_4_ | Organoheterocyclic compounds | 36059 |
| S_met_33 | Auroxanthin | C_40_H_56_O_4_ | Lipids and lipid-like molecules | 30478 |
| S_met_87 | Isoproturon-monodemethyl | C_11_H_16_N_2_O | Benzenoids | 29050 |
| S_met_112 | PFCA-perfluoroalkyl_Hsubstituted_1DB | - | - | 28998 |

^1^Annotations matching human pharmaceuticals/steroids/controlled substances (e.g., morphine and related entries) are probable isobaric misannotations of plant/soil-derived isomers and are excluded from biological interpretation (Section 4.6).

**Supplementary Table S2.** Rhizosphere-soil (RS)-Top 25 exclusive features, with putative annotations^1^

| **Metabolite ID** | **Metabolite Nomenclature** | **Molecular formula** | **Metabolite Superclass** | **RS Relative Abundance** |
| --- | --- | --- | --- | --- |
| S_met_239 | delta-Tridecalactone | C_13_H_24_O_2_ | Organoheterocyclic compounds | 160457 |
| S_met_214 | N, N-Diethyl-4-methoxybenzamide | C_12_H_17_NO_2_ | Benzenoids | 126758 |
| S_met_206 | Kaempferide | C_16_H_12_O_6_ | Phenylpropanoids and polyketides | 78361 |
| S_met_216 | alpha-Estradiol | C_18_H_24_O_2_ | Lipids and lipid-like molecules | 74617 |
| S_met_211 | Progesterone | C_21_H_30_O_2_ | Lipids and lipid-like molecules | 68797 |
| S_met_203 | Dexamethasone | C_22_H_29_FO_5_ | Lipids and lipid-like molecules | 67620 |
| S_met_221 | Cymoxanil | C_7_H_10_N_4_O_3_ | Organic nitrogen compounds | 63869 |
| S_met_215 | Uridine | C_9_H_12_N_2_O_6_ | Nucleosides, nucleotides, and analogues | 57349 |
| S_met_217 | 10,11-trans-Dihydroxy-10,11-dihydrocarbamazepine | C_15_H_14_N_2_O_3_ | Organoheterocyclic compounds | 54481 |
| S_met_231 | Peonidin-3,5-O-di-beta-glucopyranoside | C_28_H_33_O_16_^+^ | Phenylpropanoids and polyketides | 53006 |
| S_met_200 | Conessine | C_24_H_40_N_2_ | Lipids and lipid-like molecules | 52007 |
| S_met_213 | Matrine | C_15_H_24_N_2_O | Alkaloids and derivatives | 47116 |
| S_met_204 | FucGlcNAcGA | C_16_H_27_NO_11_ | Organic oxygen compounds | 47012 |
| S_met_232 | Primidone | C_12_H_14_N_2_O_2_ | Organoheterocyclic compounds | 43049 |
| S_met_208 | Metribuzin-diketo | C_7_H_12_N_4_O_2_ | Organoheterocyclic compounds | 40985 |
| S_met_218 | 4-Pyridoxic acid | C_8_H_9_NO_4_ | Organoheterocyclic compounds | 40641 |
| S_met_207 | L-Glutathione (reduced form) | C_10_H_17_N_3_O_6_S | Organic acids and derivatives | 37622 |
| S_met_223 | FT-sulfone | - | - | 37581 |
| S_met_237 | Veratrosine | C_33_H_49_NO_7_ | Benzenoids | 33716 |
| S_met_238 | delta-Dodecalactone | C_12_H_22_O_2_ | Organoheterocyclic compounds | 33030 |
| S_met_202 | D, L-Sulforaphane | C_6_H_11_NOS_2_ | Organosulfur compounds | 28574 |
| S_met_227 | L-Oxonoreleagnine | C_11_H_10_N_2_O | Organoheterocyclic compounds | 27171 |
| S_met_212 | Clarithromycin | C_38_H_69_NO_13_ | Organic oxygen compounds | 26470 |
| S_met_228 | NP-000062(6) | C_26_H_28_O_14_ | Phenylpropanoids and polyketides | 26336 |
| S_met_201 | Crystal violet | C_25_H_30_ClN_3_ | Benzenoids | 26162 |

^1^Annotations matching human pharmaceuticals/steroids/controlled substances are probable isobaric misannotations of plant/soil-derived isomers and are excluded from biological interpretation.

**Supplementary Table S3.** Soybean Root Sterilised (SRS) and Soybean Root Unsterilised (SRU)-Top 25 exclusive root features, with putative annotations^1^

| **Samples** | **Metabolite ID** | **Metabolite Nomenclature** | **Molecular Formula** | **Metabolite Superclass** | **Metabolite Relative Abundance** |
| --- | --- | --- | --- | --- | --- |
| **SRS**  **exclusive** | R_met_267 | L-Arginine | C_6_H_14_N_4_O_2_ | Organic acids and derivatives | 38999 |
|  | R_met_154 | Gentiobiose | C_12_H_22_O_11_ | Organic oxygen compounds | 28056 |
|  | R_met_342 | Prednisone | C_21_H_26_O_5_ | Lipids and lipid-like molecules | 26986 |
|  | R_met_249 | Histidine | C_6_H_9_N_3_O_2_ | Organic acids and derivatives | 25445 |
|  | R_met_196 | Chrysanthemic Acid | C_10_H_16_O_2_ | Lipids and lipid-like molecules | 23825 |
|  | R_met_181 | Brefeldin-A | C_16_H_24_O_4_ | Phenylpropanoids and polyketides | 21866 |
|  | R_met_234 | Galaxolidone | C_18_H_24_O_2_ | Organoheterocyclic compounds | 20724 |
|  | R_met_176 | Avocadyne Acetate | C_19_H_34_O_4_ | Lipids and lipid-like molecules | 19484 |
|  | R_met_250 | Hydrocortisone | C_21_H_30_O_5_ | Lipids and lipid-like molecules | 17455 |
|  | R_met_215 | Desoxypeganine | C_11_H_12_N_2_ | Organoheterocyclic compounds | 16530 |
|  | R_met_333 | PFSM-perfluoroalkyl_sulfonamide_Et; C4H6F5NO2S | C_4_H_6_F_5_NO_2_S | Organic acids and derivatives | 15418 |
|  | R_met_129 | 4-hydroxyphenylacetic acid | C_8_H_8_O_3_ | Benzenoids | 12932 |
|  | R_met_284 | Lobeline Hydrochloride | C_22_H_27_NO_2_ | Organic oxygen compounds | 12406 |
|  | R_met_301 | Methyl glucosyl-3,4-dehydro-apo-8'-lycopenate | C_37_H_52_O_8_ | Lipids and lipid-like molecules | 11992 |
|  | R_met_77 | 2,3,12-trihydroxy-4,6a,6b,11,12,14b-hexamethyl-8a-[3,4,5-trihydroxy-6-(hydroxymethyl)oxan-2-yl]oxycarbonyl-1,2,3,4a,5,6,7,8,9,10,11,12a,14,14a-tetradecahydropicene-4-carboxylic acid | C_36_H_56_O_12_ | Lipids and lipid-like molecules | 10764 |
|  | R_met_78 | 2,5-DIHYDROXYBENZOIC ACID | C_7_H_6_O_4_ | Benzenoids | 7774 |
|  | R_met_264 | Koparin | C_16_H_12_O_6_ | Phenylpropanoids and polyketides | 7622 |
|  | R_met_182 | CHOLIC ACID | C_24_H_40_O_5_ | Lipids and lipid-like molecules | 7575 |
|  | R_met_259 | Isosakuranetin | C_16_H_14_O_5_ | Phenylpropanoids and polyketides | 5988 |
|  | R_met_136 | 5-[5-hydroxy-3-(hydroxymethyl)pentyl]-8a-(hydroxymethyl)-5,6-dimethyl-3,4,4a,6,7,8-hexahydronaphthalene-1-carboxylic acid | C_20_H_34_O_5_ | Lipids and lipid-like molecules | 5312 |
|  | R_met_387 | [(3R,4S,8S,8aS)-3-hydroxy-6,8a-dimethyl-8-[(Z)-2-methylbut-2-enoyl]oxy-3-propan-2-yl-1,2,3a,4,5,8-hexahydroazulen-4-yl] 4-hydroxybenzoate | C_27_H_36_O_6_ | Lipids and lipid-like molecules | 4965 |
|  | R_met_379 | [(1R,2S,3S,4R,4aS)-2,3-dihydroxy-3,4a,8,8-tetramethyl-4-[(E)-3-oxobut-1-enyl]-2,4,5,6,7,8a-hexahydro-1H-naphthalen-1-yl] acetate | C_20_H_32_O_5_ | Lipids and lipid-like molecules | 4301 |
|  | R_met_367 | Thymol | C_10_H_14_O | Lipids and lipid-like molecules | 4265 |
|  | R_met_291 | Matairesinol | C_20_H_22_O_6_ | Lig-ns, neolig-ns and related compounds | 3923 |
|  | R_met_364 | Tebufenozide | C_22_H_28_N_2_O_2_ | Benzenoids | 3507 |
| **SRU exclusive** | R_met_400 | arthrobactin | C_20_H_36_N_4_O_9_ | Lipids and lipid-like molecules | 1130136 |
|  | R_met_199 | Citrinin | C_13_H_14_O_5_ | Organoheterocyclic compounds | 175654 |
|  | R_met_125 | 4-[5-[[4-[5-[acetyl(hydroxy)amino]pentylamino]-4-oxobutanoyl]-hydroxyamino]pentylamino]-4-oxobutanoic acid | C_20_H_36_N_4_O_8_ | Lipids and lipid-like molecules | 125301 |
|  | R_met_308 | N-(9-oxodecyl)acetamide | C_12_H_23_NO_2_ | Organic acids and derivatives | 112929 |
|  | R_met_115 | 3-hydroxy-8-methoxy-3-methyl-2,4-dihydrobenzo[a]anthracene-1,7,12-trione | C_20_H_16_O_5_ | Phenylpropanoids and polyketides | 83235 |
|  | R_met_324 | Ouabain | C_29_H_44_O_12_ | Lipids and lipid-like molecules | 75397 |
|  | R_met_104 | 3,5-dihydroxy-2-(4-hydroxyphenyl)-6-(3-methylbut-2-enyl)-7-[3,4,5-trihydroxy-6-(hydroxymethyl)oxan-2-yl]oxy-2,3-dihydrochromen-4-one | C_26_H_30_O_11_ | Phenylpropanoids and polyketides | 72061 |
|  | R_met_131 | 5-(1,2,3,4,5,6-hexahydroxyhexoxy)-6-hydroxy-3-methoxy-9-methyl-4,5-dihydro-3H-pyrano[3,4-h][1]benzoxepine-2,7-dione | C_21_H_26_O_13_ | Organoheterocyclic compounds | 70193 |
|  | R_met_179 | Bavachinin A | C_21_H_22_O_4_ | Phenylpropanoids and polyketides | 69407 |
|  | R_met_413 | eupomatenoid 5 | C_19_H_18_O_3_ | Phenylpropanoids and polyketides | 52853 |
|  | R_met_383 | [(2R,3R,4S,5S,6R)-3-acetyloxy-2-(acetyloxymethyl)-5-hexanoyloxy-6-[(2S,3R)-2,3,4-trihydroxybutoxy]oxan-4-yl] tetradecanoate | C_34_H_60_O_13_ | Lipids and lipid-like molecules | 50289 |
|  | R_met_211 | Deacetylgedunin | C_26_H_32_O_6_ | Lipids and lipid-like molecules | 47773 |
|  | R_met_108 | 3-Deoxy-3Beta-Hydroxyangolensic Acid Methyl Ester | C_27_H_36_O_7_ | Organoheterocyclic compounds | 43311 |
|  | R_met_63 | 10'-Apozeaxanthinal | C_27_H_36_O_2_ | Lipids and lipid-like molecules | 39709 |
|  | R_met_198 | Citric acid | C_6_H_8_O_7_ | Organic acids and derivatives | 37794 |
|  | R_met_260 | Isotalatizidine | C_23_H_37_NO_5_ | Lipids and lipid-like molecules | 35270 |
|  | R_met_3 | (1R,2R,4S,7R,8S,12R)-7-(furan-3-yl)-1,8,12,17,17-pentamethyl-3,6,16-trioxapentacyclo[9.9.02,4.02,8.012,18]icos-13-ene-5,15,20-trione | C_26_H_30_O_7_ | Lipids and lipid-like molecules | 34964 |
|  | R_met_302 | Methylprednisolone | C_22_H_30_O_5_ | Lipids and lipid-like molecules | 34113 |
|  | R_met_185 | CRUSTECDYSONE | C_27_H_44_O_7_ | Lipids and lipid-like molecules | 33695 |
|  | R_met_246 | Harmine | C_13_H_12_N_2_O | Alkaloids and derivatives | 33297 |
|  | R_met_380 | [(1S,2R,4aR,8aR)-1-acetyloxy-1,4a-dimethyl-6-oxo-7-propan-2-ylidene-2,3,4,5,8,8a-hexahydronaphthalen-2-yl] 3-acetyloxy-2-hydroxy-2-methylbutanoate | C_24_H_36_O_8_ | Lipids and lipid-like molecules | 32430 |
|  | R_met_27 | (2S,3S,4S,5R,6R)-6-[[(3S,6aR,6bS,8aS,14bR)-8a-carboxy-4,4,6a,6b,11,11,14b-heptamethyl-1,2,3,4a,5,6,7,8,9,10,12,12a,14,14a-tetradecahydropicen-3-yl]oxy]-3,4,5-trihydroxyoxane-2-carboxylic acid | C_36_H_56_O_9_ | Lipids and lipid-like molecules | 29015 |
|  | R_met_376 | Xanthosine | C_10_H_12_N_4_O_6_ | Nucleosides, nucleotides, and a-logues | 28024 |
|  | R_met_363 | TRYPTOPHAN | C_11_H_12_N_2_O_2_ | Organoheterocyclic compounds | 26465 |

^1^Annotations matching human pharmaceuticals/steroids/controlled substances are probable isobaric misannotations of plant/soil-derived isomers and are excluded from biological interpretation.

**Supplementary Table S4.** Large-fold-change candidate and FDR-significant features from the SRS vs SRU comparison (including R_met_88, R_met_34 and R_met_365, which met the FDR threshold, see Table 2, and additional large-fold-change features, including daidzein (R_met_208), jasmonic acid (R_met_1), and OPDA (R_met_69), that did not), with raw and adjusted p-values."

| **Met ID** | **Metabolite Name** | **Metabolite Superclass** | **p-value** | **Adjusted p-value** | **Difference of Means (log₂ FC)** |
| --- | --- | --- | --- | --- | --- |
| R_met_1 | (+-)-Jasmonic acid | Lipids and lipid-like molecules | 0.025 | 0.254 | -6.76 |
| R_met_14 | (2E,4E)-1-[(2R,6S,14S,22S,25R)-25-(3,3-dimethyloxiran-2-yl)-15-methyl-1,3,13,15-tetraazaheptacyclo[18.4.1.0.0.0.0.0]pentacosa-7,9,11,16(21),17,19-hexaen-3-yl]hexa-2,4-dien-1-one | Organoheterocyclic compounds | 0.036 | 0.254 | -2.682 |
| R_met_30 | (3,5,9,13,16,17,22R,23R,24R)-3-{[L-Arabinofuranosyl-(1->2)-[β-D-glucopyranosyl-(1->3)]-α-L-arabinopyranosyl]oxy}-20-hydroxy-24-methyl-16,23:16,30-diepoxydammar-25-en-22-yl acetate | Lipids and lipid-like molecules | 0.002 | 0.0526 | 8.492 |
| R_met_31 | (3,8,9,24E)-3-[(6-O-Acetyl-4-O-pentopyranosylhexopyranosyl)oxy]-12,16-dihydroxy-9,19-cyclolanost-24-en-26-oic acid | Lipids and lipid-like molecules | 0.044 | 0.254 | 4.391 |
| R_met_34 | (3S,4S,6aR,6bS,8R,8aR,12aS,14bR)-8-hydroxy-4,6a,6b,11,11,14b-hexamethyl-3-[(2S,3R,4S,5R)-3,4,5-trihydroxyoxan-2-yl]oxy-1,2,3,4a,5,6,7,8,9,10,12,12a,14,14a-tetradecahydropicene-4,8a-dicarboxylic acid | Lipids and lipid-like molecules | 0.001 | 0.026 | -7.442 |
| R_met_42 | (9Z,12E)-15,16-dihydroxyoctadeca-9,12-dienoic acid | Lipids and lipid-like molecules | 0.0464 | 0.254 | +4.88 |
| R_met_62 | 1-palmitoyl-2-oleoyl-sn-glycero-3-phosphocholine | Lipids and lipid-like molecules | 0.031 | 0.254 | 2.791 |
| R_met_69 | 12-Oxo-10,15(Z)-Phytodienoic Acid (OPDA) | Lipids and lipid-like molecules | 0.0901 | 0.327 | +4.10 |
| R_met_82 | 2-(7-methoxy-2-oxochromen-8-yl)-3-methylbut-2-enal | Phenylpropanoids and polyketides | 0.046 | 0.254 | 1.494 |
| R_met_88 | 2-[(6,7-dimethoxy-1,2,3,4-tetrahydroisoquinolin-1-yl)methyl]-3-ethyl-9,10-dimethoxy-5-oxido-2,3,4,6,7,11b-hexahydro-1H-benzo[a]quinolizin-5-ium | Alkaloids and derivatives | 4.409 × 10^-4^ | 0.0257 | -10.831 |
| R_met_90 | 2-[2,6-bis(3-methylbut-2-enyl)-4-[(2S,3R,4S,5S,6R)-3,4,5-trihydroxy-6-(hydroxymethyl)oxan-2-yl]oxyphenyl]-2-hydroxyacetic acid | Organic oxygen compounds | 0.007 | 0.125 | -7.972 |
| R_met_101 | 3,4,5-trihydroxy-6-(hydroxymethyl)oxan-2-yl (2E,6R)-6-[(1R,3R,6S,8R,12S,15R,16R)-13,17-dihydroxy-7,7,12,16-tetramethyl-6-{[(2R)-3,4,5-trihydroxy-6-{[(3,4,5-trihydroxyoxan-2-yl)oxy]methyl}oxan-2-yl]oxy}pentacyclo[9.7.0.0.0.0]octadecan-15-yl]-2-methylhept-2-enoate | Lipids and lipid-like molecules | 0.006 | 0.125 | -9.269 |
| R_met_183 | Cinnamaldehyde | Phenylpropanoids and polyketides | 0.032 | 0.254 | -3.802 |
| R_met_208 | Daidzein | Phenylpropanoids and polyketides | 0.034 | 0.254 | 3.410 |
| R_met_258 | Isosafrole | Organoheterocyclic compounds | 0.014 | 0.222 | 4.363 |
| R_met_283 | Linoleic Acid | Lipids and lipid-like molecules | 0.021 | 0.254 | 1.516 |
| R_met_293 | Menaquinone-4 | Lipids and lipid-like molecules | 0.026 | 0.254 | -3.030 |
| R_met_321 | Orth0-Aminobenzoic Acid | Benzenoids | 0.022 | 0.254 | 1.221 |
| R_met_351 | Saikosaponin a | Lipids and lipid-like molecules | 0.006 | 0.125 | 1.744 |
| R_met_358 | Spegatrine | Alkaloids and derivatives | 0.035 | 0.254 | 5.807 |
| R_met_365 | Terfenadine | Benzenoids | 0.001 | 0.026 | -2.414 |
| R_met_369 | Trimipramine | Organoheterocyclic compounds | 0.048 | 0.254 | 2.409 |
| R_met_390 | [(7R,8R,10R,13S)-17-(furan-3-yl)-4,4,8,10,13-pentamethyl-3,16-dioxo-6,7,9,11,12,17-hexahydro-5H-cyclopenta[a]phenanthren-7-yl] acetate | Lipids and lipid-like molecules | 0.042 | 0.254 | -2.799 |
| R_met_397 | Albocycline | Phenylpropanoids and polyketides | 0.038 | 0.254 | 4.105 |
| R_met_429 | Rubiadin | Benzenoids | 0.039 | 0.254 | -4.950 |

**Supplementary Figure Legends**

**Supplementary Fig. S1.** Three-dimensional PCA score plots (NRBS/RS and SRU/SRS).

**Supplementary Fig. S2.** Heatmap/hierarchical clustering, NRBS vs RS.

**Supplementary Fig. S3.** VIP scores, NRBS vs RS.

**Supplementary Fig. S4.** Heatmap/hierarchical clustering, SRS vs SRU.

**Supplementary Fig. S5.** VIP scores, SRS vs SRU.

**Fig.S1.**

**Fig. S2**

**Fig. S3**

**Fig. S4**

**Fig. S5**
